# Insecticide-treated net distribution in Western Kenya: impacts related to COVID-19 and health worker strikes

**DOI:** 10.1093/inthealth/ihab051

**Published:** 2021-08-16

**Authors:** Laurissa Suiyanka, Victor A Alegana, Robert W Snow

**Affiliations:** Population Health Unit, Kenya Medical Research Institute-Wellcome Trust Research Programme, P.O. Box 43640-00100, Nairobi, Kenya; Population Health Unit, Kenya Medical Research Institute-Wellcome Trust Research Programme, P.O. Box 43640-00100, Nairobi, Kenya; Geography and Environmental Science, University of Southampton, University Road, Southampton, SO17 1BJ, UK; Population Health Unit, Kenya Medical Research Institute-Wellcome Trust Research Programme, P.O. Box 43640-00100, Nairobi, Kenya; Centre for Tropical Medicine and Global Health, Nuffield Department of Clinical Medicine, University of Oxford, Oxford, OX3 7LJ, UK

**Keywords:** Covid-19, LLIN, Malaria

## Abstract

We examined the impact of coronavirus disease (COVID) mitigation, supply and distribution interruptions on the delivery of long-lasting insecticide-treated nets (LLINs) in Western Kenya. The median monthly distribution of LLINs declined during COVID mitigation strategies (March–July 2020) and during the health worker strikes (December 2020–February 2021). Recovery periods followed initial declines, indicative of a ‘catching up’ on missed routine distribution. Mass community campaigns were delayed by 10 months. These observations offer encouragement for routine net distribution resilience, but complete interruptions of planned mass distributions require alternate strategies during pandemics.

## Introduction

Sudden acute respiratory syndrome coronavirus 2 (SARS-CoV-2), a novel coronavirus, emerged in 2019 and has impacted health, livelihoods and access to basic services globally. The precise disease burden experienced in sub-Saharan Africa (SSA) remains unknown. Beyond the direct consequences of coronavirus disease 2019 (COVID-19) infection, interruptions in the routine delivery of health services in low- and middle-income countries is predicted to have a large indirect impact on population health.^1^

**Figure 1.**
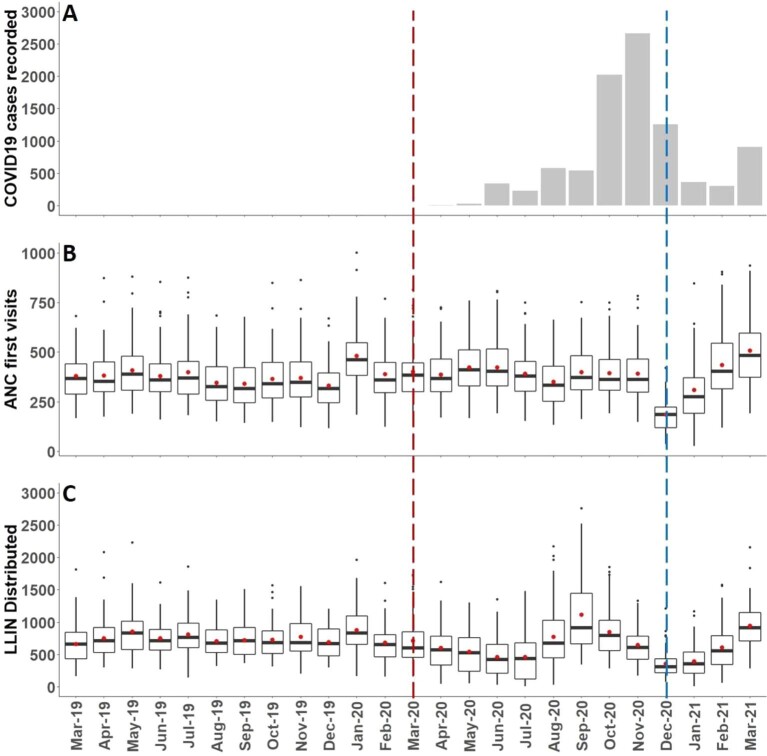
The temporal impact of health system shocks on routine public health service attendance and distribution on LLINs, March 2019–March 2021. (**A**) Health system shocks: COVID mitigation (red dotted line) and health worker strikes (blue dotted line) and reported COVID-positive tests in Western Kenya. (**B**) Monthly ANC attendance and (**C**) monthly distributions of LLINs to children and pregnant women averaged across 62 subcounties to capture spatial variability in service provision across the 8 counties in Western Kenya from aggregated data recorded March 2019–March 2021 at 1181 public health facilities where routine LLINs are distributed. Box-and-whisker plots represent the median (dark mid-box line) and interquartile 25th and 75th percentile range (box width). The mean is shown as red dots, outliers in the data are represented by black dots. Overall reporting rates across all subcounties during for the 29 525 possible facility months was 94% for ANC first attendance, 85% for ANC distributions of LLINs and 81% for child welfare/immunisations. No significant differences were observed between counties or reporting periods.

The first case of COVID-19 was detected in Kenya on 13 March 2020. National COVID-19 mitigation protocols were announced between 22 March and 19 April 2020 that included curfews; mandated wearing of facemasks; closure of schools, higher education institutions, bars, restaurants and places of worship; and bans on international travel and transport (Figure 1A). Between 6 May and 7 June 2020, the capital city of Nairobi and the seaport of Mombasa were placed under lockdown, preventing movement in and out of these urban centres.^2^ Restrictions were relaxed between August and November 2020.^2^ A second major lockdown was announced on 26 March 2021, following a third wave of increasing COVID-19 test-positivity, reinstating earlier restrictions and the lockdown of travel into and out of Nairobi and four neighbouring counties.

Health worker strikes, involving different cadres of staff in different locations, lasting between 3 and 7 d, occurred during 2019 and in December 2020 due to pay disputes, contracts and COVID safety concerns, including availability of personal protective equipment. However, health workers returned to their jobs by mid-February 2021.

## Methods

We examined the impact of health system interruptions on the delivery of long-lasting insecticide-treated nets (LLINs) between March 2019 and March 2021 using temporally and spatially assembled routine health information system data from 62 subcounties that cover eight counties surrounding Lake Victoria in Kenya (Homa Bay, Kisumu, Migori, Vihiga, Siaya, Bungoma, Busia and Kakamega). The area represents the highest malaria transmission counties in Kenya^3^ and has been the focus of malaria prevention as part of a subnational, stratified control response, including ensuring high coverage of LLINs, since 2010.^4^

LLINs are provided free of charge to all pregnant women at their first antenatal care (ANC) visit and to all children as they attend welfare and vaccination clinics, which also makes it difficult to account for double allocation of LLINs. In addition, mass household distribution campaigns are used to maximise household coverage. The last mass campaigns were undertaken between June and November 2017, distributing 6.2 million LLINs to residents of the eight counties. The next community campaign was planned to start in June 2020 but was cancelled due to COVID and started in April 2021.

The District Health Information System version 2 (DHIS2) serves as the national data platform for health service delivery evaluation. Monthly counts of LLINs provided to children and ANC attendees were extracted per public health facility from the DHIS2 platform covering 25 months from March 2019 to March 2021. To understand potential attendance for routine services, monthly counts of ANC first visits were also extracted per facility for the same period. Data were summarized monthly across all reporting facilities and expressed as means, medians and interquartile ranges (IQRs) to reflect the variability in service use and provision (Figure 1B and C).

The median monthly subcounty distribution of LLINs to children attending routine health checks and immunization services or pregnant women attending ANC prior to the COVID mitigation strategy introduction in March 2020 was 730 (IQR 539–944). Thereafter, the monthly distributions progressively decreased from April to July 2020 (Figure 1C), a result of import delays at the port of Mombasa due to tax levies, compounded by subsequent supply interruptions to central stores in Nairobi and then to counties; however, this was against a sustained constant service access as defined by ANC attendance (Figure 1B). A catch-up was observed from August to October 2020, when resupply was re-established and appeared to fill a previous unfulfilled distribution (Figure 1C). During the period of potential impacts of COVID on routine service provision of LLINs (March–November 2020), and prior to the national health worker strike, the median monthly subcounty distribution volume was 615 (IQR 388–893). The health workers strike impacted significantly on both health service attendance (Figure 1B) and the distribution of LLINs from December 2020 to February 2021 (Figure 1C), however, catch-up was observed for both first ANC attendance and LLIN distribution when the strike ended. For this last period (December 2020–March 2021), the median monthly subcounty LLIN distribution volume was 527 (IQR 294–798).

### Conclusions

The effects of the COVID pandemic have had multiple, connected impacts on the distribution of LLINs in Western Kenya: mass community-based campaigns were delayed; supply chains were affected, leading to temporary declines in routine distributions; and COVID-related health worker strikes disrupted healthcare delivery. Supply and COVID-related health system shocks during the first and second waves of infection did not completely interrupt access to LLINs, were temporary and showed signs of resilience by ‘catching up’ on missed distribution opportunities. It is important to understand the complexities of crises on national malaria vector control, building empirical knowledge for lessons on bottlenecks, consequential impacts (strikes) and system resilience to map future scenarios.

## Data Availability

Aggregated DHIS2 data are available online with access provided by the Ministry of Health (https://hiskenya.org/dhis-web-commons/security/login.action). The datasets used and/or analysed during the current study are available from the corresponding author on reasonable request.
